# Cellulose Nanocrystal Embedded Composite Foam and Its Carbonization for Energy Application

**DOI:** 10.3390/polym15163454

**Published:** 2023-08-18

**Authors:** So Yeon Ahn, Chengbin Yu, Young Seok Song

**Affiliations:** 1 Department of Fiber Convergence Materials Engineering, Dankook University, Jukjeon-dong, Yongin 16890, Republic of Korea; soyeon@dankook.ac.kr; 2 Research Institute of Advanced Materials (RIAM), Department of Materials Science and Engineering, Seoul National University, Seoul 08826, Republic of Korea

**Keywords:** cellulose nanocrystal, chitosan, carbonized structure, voltammetric

## Abstract

In this study, we fabricated a cellulose nanocrystal (CNC)-embedded aerogel-like chitosan foam and carbonized the 3D foam for electrical energy harvesting. The nanocrystal-supported cellulose foam can demonstrate a high surface area and porosity, homogeneous size ranging from various microscales, and a high quality of absorbing external additives. In order to prepare CNC, microcrystalline cellulose (MCC) was chemically treated with sulfuric acid. The CNC incorporates into chitosan, enhancing mechanical properties, crystallization, and generation of the aerogel-like porous structure. The weight percentage of the CNC was 2 wt% in the chitosan composite. The CNC/chitosan foam is produced using the freeze-drying method, and the CNC-embedded CNC/chitosan foam has been carbonized. We found that the degree of crystallization of carbon structure increased, including the CNCs. Both CNC and chitosan are degradable materials when CNC includes chitosan, which can form a high surface area with some typical surface-related morphology. The electrical cyclic voltammetric result shows that the vertical composite specimen had superior electrochemical properties compared to the horizontal composite specimen. In addition, the BET measurement indicated that the CNC/chitosan foam possessed a high porosity, especially mesopores with layer structures. At the same time, the carbonized CNC led to a significant increase in the portion of micropore.

## 1. Introduction

Sustainable development is a top issue in human life, and sustainable and renewable materials are utilized in many application fields [1,2]. The carbon-based materials are selected as fillers to improve the availability of polymer composites [3,4]. Some natural polymers, such as cellulose, chitin, silk, wool, and protein, are primarily water-based and extracted in nature [5,6]. It is a green energy selection for cellulose-based composites [7,8]. For instance, since cellulose has many strong points, such as low cost, good chemical and physical properties, and low environmental load, it has attracted significant attention in material science and engineering [9,10]. In addition, cellulose aerogel can be applied in medical areas, which requires biocompatibility and biodegradability [11,12]. The aerogel produces using cellulose and cellulose derivatives. For example, since nano-fibrillated cellulose (NFC) is a cellulosic nanomaterial with good crystallinity and sizeable specific surface due to the cellulose I structure, it can serve as a structural material for the aerogel [13,14]. Although cellulose can extend its applications, its relatively low mechanical property must be improved significantly [15,16]. On the other hand, much effort has been made to construct a form-stable cellulose compound for energy-related applications by carbonizing cellulosic materials [17,18]. The carbonization of cellulose generally needs to use catalysts or surfactants to promote cellulose transformation at high temperatures and pressure [19,20]. Considering the abundance and cost of cellulose, embedding and carbonizing cellulose can contribute to expanding applications related to carbonized materials [21,22]. Since carbon is one of the most popular materials for electrodes, energy storage materials, and catalysts, the carbonization of natural organic materials such as cellulose has a high potential [23,24]. Furthermore, cellulose particles behave electro-rheologically and can control temporally and spatially using an electric field [25,26]. In the energy harvesting area, carbon is a fascinating material for promoting electron transfer and providing ion storage [27,28]. However, polarized electrical particles disperse irregularly, and that large portion is complete in the non-conducting area due to the absence of electric fields [29,30]. The rigid chain structures formed by the particle arrangement limit the electrical energy harvesting applications [31,32].

Chitosan is widely utilized as a renewable polymer in various applications, such as medical, drug delivery, and tissue engineering [33,34]. It can quickly decompose because of its biodegradable structure, making chitosan a renewable material for various applications [35,36]. Furthermore, chitosan is non-toxic and potentially valuable for drug delivery and green energy development [37]. The chemical structure of chitosan merely includes a primary amine (−NH_2_) group, and it can adsorb certain kinds of molecules from the aqueous solutions [38,39]. Therefore, chitosan can exhibit intrinsic physical and chemical properties such as non-toxicity, excellent antimicrobial performance, and membrane-associated performance [40,41]. Cellulose aerogel is a porous, lightweight, and flexible material made from cellulose according to the chemical structure (e.g., sugar moieties). It can be a promising agent for targeting some chemical components in bioengineering. Since chitosan has plenty of amine and hydroxyl functional groups, various chemical and physical treatments for it, such as hydrolysis, cross-linking, and polymerization, can be employed for further applications [42,43]. For example, chitosan derivatives can be utilized in electrical energy storage and harvesting applications [44,45] since the chitosan foam is created by adding a foaming agent and evaporating the solvent, which can solidify to an aerogel-like structure [46]. The aerogel-like chitosan foam can break down under external conditions, and this property causes a chance to modify a range of applications [47,48]. The porous chitosan foam is utilized as a power generator due to the continuous skeletons in the porous structure, which performs as an electron carrier to increase the electrical conductivity [49,50]. It is well known that the phase change material (PCM) with a high thermal energy storage (TES) is broadly utilized for thermoelectric energy harvesting due to the Seebeck effect [51,52]. The phase transition process gave rise to the temperature difference at two sides of the thermoelectric power generator (TEG) and induced electron movement in the closed circuit [53,54]. In order to prevent the PCM leakage problem, 3D porous supporting materials such as graphene and silica aerogels are employed to infiltrate pure PCM for fabricating the form-stable PCM composite [54,55]. In this research area, the porous aerogels can maintain the solid state of PCM composite without any leakage during the melting process and increase both thermal and electrical conductivities significantly [56,57]. The pore size distribution seems to control the foam porosity, mechanical strength, and electron.

Based on the typical properties of cellulose and chitosan, the cellulose-incorporated chitosan composites were characterized, and obtained functional structures were evaluated. The cellulose nanocrystal (CNC), glutaraldehyde (Glu), and chitosan ternary composites were fabricated with different CNC/chitosan ratios and characterized using various techniques [58]. The composite showed an emulsion state, and the internal crystal size was affected by pH and temperature variations. This study mentioned that cellulose nanocrystal (CNC)-modified chitosan composite could improve the emulsifying capacity and crystal structures significantly. The composite under the emulsion state was difficult to fabricate as a 3D porous structure due to the immiscible substances in the aqueous solution. There was a need for constructing a porous composite with cellulose nanocrystal (CNC), and the freeze-drying process was utilized to get a sponge-like matrix [59]. It was possible to fabricate cellulose foams with different concentrations. However, different kinds of cellulose-embedded chitosan composite were prepared for an advanced nanocomposite which can reinforce the porosity and cyclic voltammetry.

Since cellulose and chitosan are natural polymers with similar chemical structures, cellulose particles such as nano-fibrillated cellulose (NFC) and cellulose nanocrystal (CNC) were incorporated into the chitosan easily. In this sense, cellulose nanoparticle-embedded chitosan composite can show enhanced physical properties such as mechanical and electrical properties [60,61]. This study embedded CNC into chitosan and composite foam preparation using freeze-drying. After that, the CNC-embedded foam carbonizes at a high temperature. The physicochemical features of the carbonized foam, such as chemical, structural, electrochemical and morphological properties, were analyzed experimentally. The carbonized foam could significantly increase electrical energy harvesting, including CNC nanoparticles.

## 2. Experimental

### 2.1. Materials

Microcrystalline cellulose (MCC) was hydrolyzed chemically. The MCC was purchased from Acros Organics. The diameter of the MCC was 50 μm and the Chitosan was provided from Sigma Aldrich. For the hydrolysis, sulfuric acid and filter paper with a pore size of 700 nm were supplied by Ducksan Chemical (Yongin, Republic of Korea) and Hyundai Micro (Seoul, Republic Korea), respectively. A cyanobacterial strain, Synechococcus, was obtained from the Korea Research Institute of Bioscience and Biotechnology (KRIBB) and grown in a BG-11 medium in an incubator with shaking at 25 °C. The microalgae were harvested on day 30. 2,5-dimethyl-1,4-benzoquinone (DMBQ), mediator was purchased from Sigma Aldrich (St. Louis, MI, USA). The platinum mesh was purchased from Ametek Inc. (Berwyn, PA, USA). MEET Co. (Seoul, Republic Korea) supplied carbon felt, and Nafion membrane was purchased from Dupont (Wilmington, DE, USA).

### 2.2. Preparation of Carbonized CNC/Chitosan Foam

**Figure 1:**
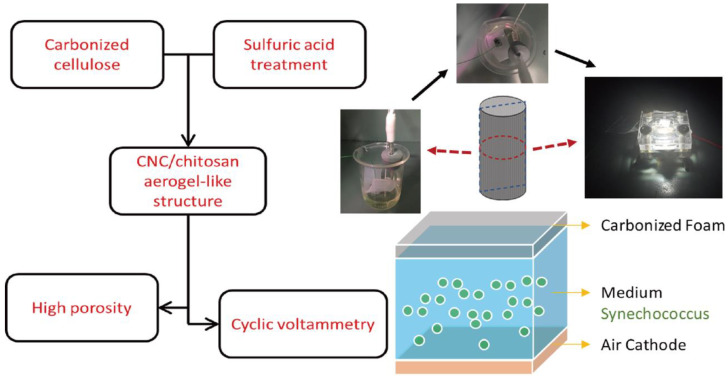
Schematic of fabrication of modified CNC/chitosan composite structure.

Figure 1 shows a brief schematic of carbonized CNC/chitosan composite foam. Before CNC/chitosan foam fabricates, CNC nanoparticles need to be prepared. First, the MCC was treated with sulfuric acid of 64 wt% at 45 °C for 2 h so that the chemical chain and particle dimension change were made under sulfuric acid. After purification, remove access sulfuric acid and prepare the CNC suspension. Since the size of CNC can affect the generation of foam structure, the treatment time and pH need to be controlled precisely. In order to increase the CNC weight fraction, the suspension rinses increased several times. Distilled water and the suspension were filtered to remove the solvent. After that, the CNC powder is produced by using the freeze-drying method. The sample dries for 48 h. Second, the CNC/chitosan suspension was prepared. The chitosan dissolves in acetic acid of 1 wt% at a 40 °C oil bath. The suspension is stirred for 2 h. Afterward, the CNC powders disperse in the chitosan solution by applying ultrasonication for 30 min, which can enhance the dispersion of the particles. In the current study, the weight fraction of the CNC was set at 2 wt% because we tried to analyze the effect of the existence of the nanoparticle in the carbonized nanoparticle-filled composite foam.

Third, the CNC/chitosan foam structure was generated. In order to do this, the CNC/chitosan solution was poured into the centrifugation tube and cooled down using the liquid nitrogen. All samples should be frozen to ice structures and put into the freeze-dryer (ilShinBioBase Co., Gyeonggi, Republic of Korea). Thereafter, the CNC/chitosan samples were under the freeze-drying process for 48 h to evaporate the solvent. The temperature at the bottom of the sample sets was lower than at the top to employ directional freezing. The temperature gap was less than 5 °C. Finally, using a furnace, the CNC/chitosan composite foam carbonizes in an argon gas environment (GTF0850, GSS, Republic of Korea). The sample was put into the furnace at 150 °C for 30 min to improve the adsorption of chitosan molecules onto the CNC surface. After that, the furnace temperature increased to 1200 °C at 10 C/min, and the specimen was for 2 h places. Finally, this specimen was placed at the room temperature to obtain an aerogel-like CNC/chitosan foam. In this study, the effect of not only the addition of the CNC particles in the foam but also the carbonization of the composite foam is investigated.

### 2.3. Characterization

An optical image of the sample surface is obtained using a microscope (Olympus SZX7, Olympus, Tokyo, Japan). A field emission scanning electron microscope was used to conduct (FESEM, S-4800, Hitachi, Hitachi, Japan) analysis. For the sample preparation, the samples were fractured and coated with platinum. For analysis, a cryogenic scanning electron microscope was employed (cryo-SEM, Mira-3 FEG, Tescan, Warrendale, PA, USA). After freezing the CNC/chitosan suspension, the suspension, at −100 °C for 10 min, was sublimed to prevent frozen water recrystallization. The additional morphological analysis uses a transmission electron microscope (TEM, JEM-200CX (JEOL, Tokyo, Japan). A droplet of the particle suspension on a 200 mesh TEM grid is used for the measurement deposits.

Fourier transform infrared spectroscopy (FT-IR) analysis was conducted with the use of an FT-IR spectrometer (Nicolet iS10, Thermo, Waltham, MA, USA) equipped with a smart diamond attenuated total reflection (ATR) accessory. A KBr-pellet method is used in the scan range of 4000–400 cm^−1^. Raman spectroscopy analysis uses a Raman spectrometer (LabRam Aramis, Horriba Jovin Yvon, Longjumeau, France). An excitation wavelength of 532 nm was employed, and the measurement wavelength was from 1000 to 3500 cm^−1^. The accumulation time was 100 s. Before the measurement, the Raman spectrometers calibrate to the silicon peak. X–ray diffraction analysis is conducted using a comprehensive angle X-ray scattering system (WAXS, D8 Discover, Bruker, Billerica, MA, USA) at 1000 μA with Cu Kα radiation (wavelength = 0.154 nm) in the 2θ range of 4.5–40° with a step interval of 0.02°.

In order to estimate the possibility of using an electrode, the electrochemical analysis of the sample was conducted using a photo-microbial solar cell (PMSC) system. Before the measurement, a Nafion membrane was treated as below: the membrane was put in a 3 wt% H_2_O_2_ and rinsed with distilled water for 30 min. After that, it is immersed in 0.5 M sulfuric acid for 1 h at 80 pa. Then, keep the membrane in distilled water. Membrane electrode assembly (MEA) fabricates for a cathode. A carbon felt was coated with platinum using a sputter and hot-pressed with the Nafion membrane at 130 °C for 1 min at a pressure of 5 MPa. The electrochemical tests, including cyclic voltammetry and chronoamperometry, were carried out using a potentiostat (VeraStat 3, Princeton Applied Research, Oak Ridge, TN, USA) [62]. The measurement was conducted in a Faraday cage using a resistance of 500 Ω and a light source.

The porous structure of sample was analyzed using the Brunauer–Emmet–Teller (BET) method. Nitrogen gas adsorption characteristics (Quantachrome NOVAe, 2000) are obtained. The measurement is conducted at a relative vapor pressure of 0.02 to 0.3 at −196 Pa. The average pore size of sample evaluates through the Barrett–Joyner–Halendar (BJH) analysis, which was adopted to determine pore volume and size with adsorption and desorption techniques.

## 3. Results and Discussion

**Figure 2:**
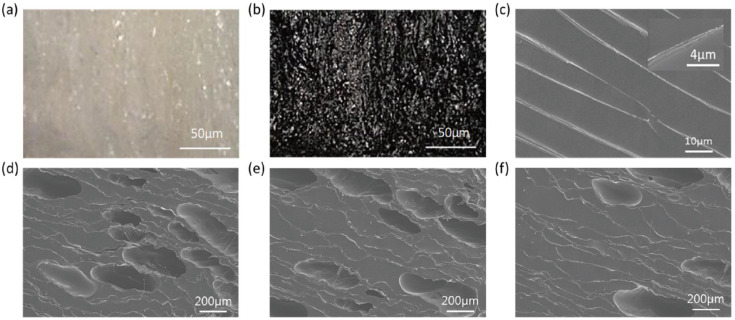
Morphological analysis of the samples: Optical microscopic images of (**a**) the CNC/chitosan foam and (**b**) the carbonized CNC/chitosan foam. (**c**) Cryo-SEM image of (**c**) the CNC/chitosan suspension and SEM images of (**d**) the carbonized chitosan foam, (**e**) the CNC/chitosan foam, and (**f**) the carbonized CNC/chitosan foam.

This study investigated how adding CNCs affects the carbonized structure since a polysaccharide nanoparticle can interact with a natural polymer. Figure 2 presents the morphological analysis results of the samples. After the CNCs were suspended in the chitosan solution, the suspension was freeze-dried (Figure 2a). All of these foam samples were prepared using the freeze-drying process. It shows that the CNCs disperse in the chitosan, generating a foam structure. In order to align the nanoparticle, which applies the directional freezing method, the directional freezing method could align the particles and crystals [63]. The directional freezing method can align the layered structure along the heat transfer direction, i.e., the cooling direction. Figure 2b represents the carbonized CNC/chitosan foam. The color turned black after carbonization, and it was modified sufficiently by the treatment.

Figure 2c shows the cryo-SEM image of the CNC/chitosan suspension. The alignment of ice layers was found, further leading to the directionality of the foam microstructure. The stripe pattern in the image was generates due to the freezing process. For this reason, the freeze-drying method used in this study can cause the anisotropic structure of the foam. Figure 2d shows the SEM image of the carbonized chitosan foam. The sample had an anisotropic porous structure. As a result of the freeze-drying method, the foam structure was formed and maintained even during carbonization. The SEM images of non-carbonized CNC/chitosan foam and carbonized CNC/chitosan foam are presented in Figure 2e,f, respectively. The CNC nanoparticles affected the carbon structure after carbonization. On the other hand, the mechanical behavior of these foams is an important physical property. The CNC/chitosan foam with cysteamine cross-linked graphene aerogel (GCA) has a similar stress–strain effect to that reported in a previous study [64].

**Figure 3:**
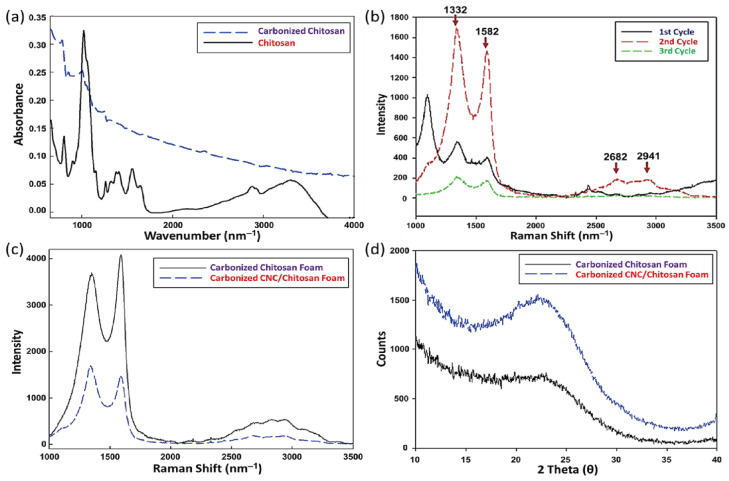
(**a**) FT-IR result of the raw and carbonized chitosan foam. (**b**) Scanning results of carbonized CNC/Chitosan foam. (**c**) Raman spectra of the carbonized chitosan foam and CNC/chitosan foam. (**d**) WAXS spectra of the carbonized chitosan foam and CNC/chitosan foam.

Figure 3a illustrates the FT-IR result of both chitosan and carbonized chitosan foams. The stretching vibration of the chitosan sample is related to the peaks between 3500 and 3250 cm^−1^, indicating the existence of O–H. The adsorption peaks of NH_2_ and secondary amides vibration of –NH are associated with peaks between 3500 and 3400 cm^−1^ and between 3300 and 3280 cm^−1^, respectively. The symmetric and asymmetric C–H vibrations induce the band of 2960–2870 cm^−1^. The C–O–C vibration indicates the 1160 cm^−1^ peak. After the carbonization, the FTIR spectra changed drastically. The C–C and C=C stretching vibrations lead to the bands at 1200 and at 1650 cm^−1^, respectively. The FT-IR peaks showed the general difference between the original and carbonized chitosan foam. The characteristic peaks of chitosan were confirmed and the complete carbonization identified. Figure 3b,c show the Raman spectra of the carbonized chitosan and CNC/chitosan foams, and Table 1 presents the corresponding typical peak results. The samples yielded graphitic D and G peaks around 1350 and 1590 cm^−1^, respectively. Furthermore, the 2D band was around 2680 cm^−1^. The shifted peak indicated the presence of highly disordered graphite and the formation of aromatic clusters. The ratio of the amount of structured carbon incorporated into the carbonized sample can be estimated using the relative intensity ratio of the D peak, and the G peak was higher than the D peak. However, once the natural nanoparticles were added into the polymer, the D peak of the composite foam showed a relatively high value. This means that the amount of amorphous carbon increases by adding the CNCs. The result of the Raman peaks indicates that the carbonized chitosan and CNC/chitosan foams had different internal structures and could affect the electrical properties.

Both carbonized chitosan foam and CNC/chitosan foam exhibited graphitic peaks. The carbonized CNC with fewer chemical functional groups had excellent chemical stability to utilize an electron carrier [65].

**Figure 4:**
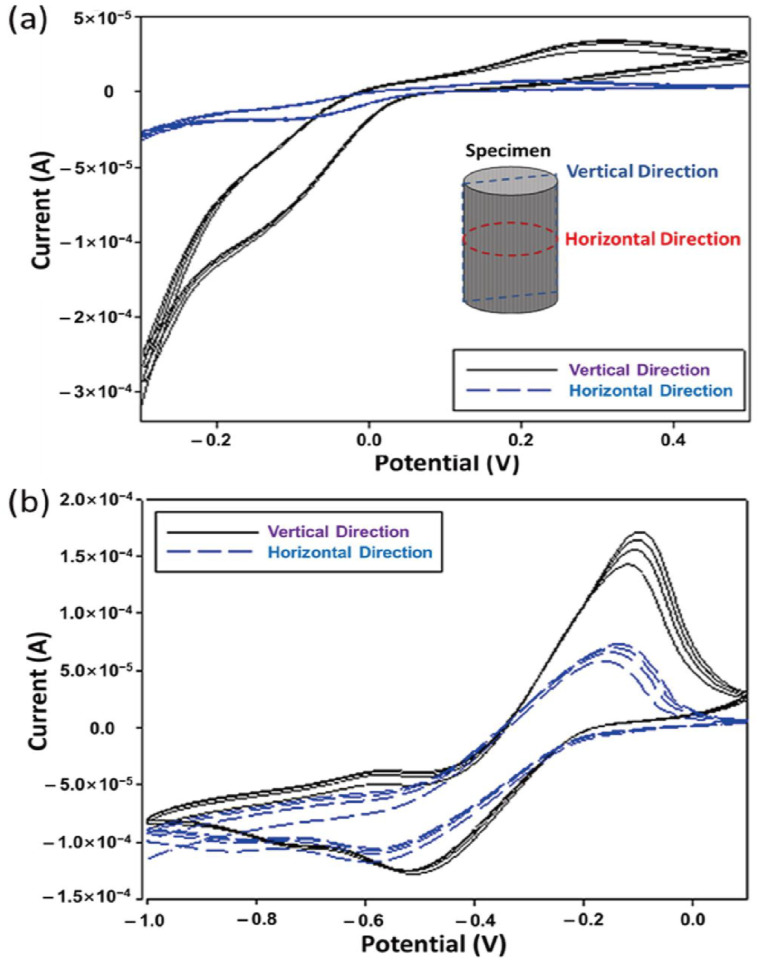
Cyclic voltammetry results of (**a**) the carbonized chitosan foam and (**b**) the carbonized CNC/chitosan foam.

**Figure 5:**
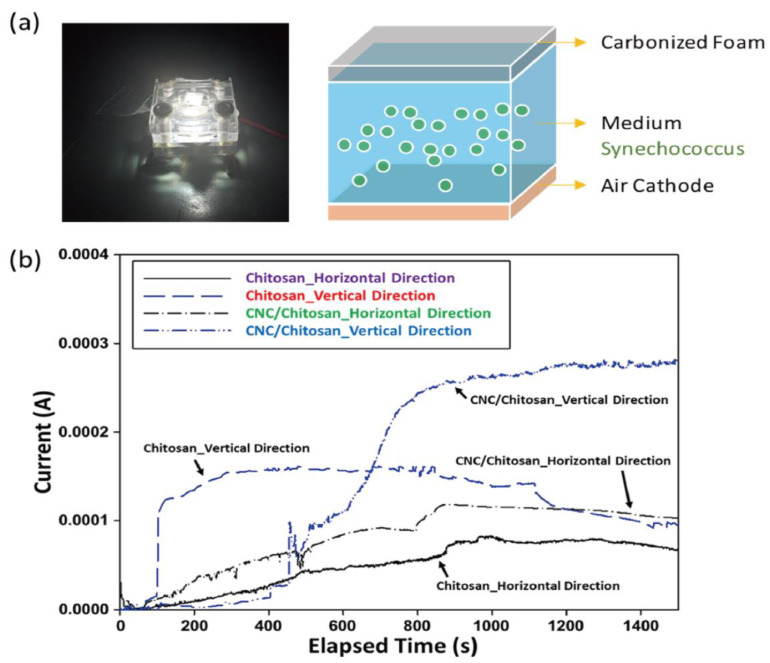
Chronoamperometry analysis of the sample: (**a**) photograph (left) and schematic image (right) of the experimental setup and (**b**) current result to time for the carbonized chitosan foam and CNC/chitosan foam.

Figure 3d presents the WAXS result of the samples. The crystal size was obtained by using the Scherrer equation: $D=\frac{K\lambda }{\beta cos\theta }$, where *D* is the size of the ordered (crystalline) domains, *K* is a shape factor, λ is the X-ray wavelength, β is the full width at half maximum (FWHM), and θ is the diffraction angle. The shape factor used in this study was 0.9, and the wavelength is 1.54 Å. The chitosan and CNC-embedded chitosan composite foams showed crystallite sizes of 0.17 and 0.13 nm, respectively. The degree of crystallization of the specimens calculated using the peak height ratio method is expressed as below:

(1) $$\chi_{CR}=\frac{I_{200}-I_{AM}}{I_{200}}\times 100$$

where $\chi_{CR}$ is the crystallinity index of a specimen, $I_{200}$ is the max intensity in 200 plane peaks, and $I_{AM}$ is the min intensity between 200 and 100 plane peaks. From the calculation results in Table 2, the crystallinity was approximately 14.0% and 24.0%, which showed the carbonized chitosan and CNC/chitosan foams, respectively. The pure chitosan specimen showed a larger crystal size but smaller crystallinity than the CNC-filled specimen. This implied that the added nanoparticle could serve as a nucleation site, thus leading to the relatively high crystal portion in the matrix. Figure 4 shows the recyclable voltammetry results of the specimens. The voltammetric behavior’s several cycles observe applied voltage and mass (or reactant) transport due to the concentration polarization and over-potential effect. It founds that the carbonized CNC/chitosan foam had a higher peak current than the carbonized chitosan foam. Note that the prepared sample possessed an anisotropic internal structure after freeze-drying, and the carbonized CNC/chitosan foam exhibited excellent recyclable behavior. Therefore, two kinds of samples were prepared for cutting in the vertical or horizontal direction, as shown in the inset. Interestingly, the vertical sample showed more extensive electrochemical characteristics than the horizontal sample. On the other hand, renewable energy harvesting is a comprehensive study to replace fossil fuel energy production. In particular, energy production using living cells, such as microbial fuel cells (MFC) and bio photovoltaic cells (BPV), is an attractive energy harvesting method. In this study, the BPV system using cyanobacteria (i.e., Synechococcus) employs to evaluate the possibility of usage as a carbon electrode. Figure 5 presents the chronoamperometry analysis result of the sample. Like the cyclic voltammetry result, the vertically prepared sample offered a higher current than the horizontally prepared sample. This indicates the existence of an anisotropic structure in the sample [66,67]. In addition, the CNC/chitosan sample possessed higher current values than the only chitosan sample due to the relatively dense internal structure of the CNC-embedded sample.

**Figure 6:**
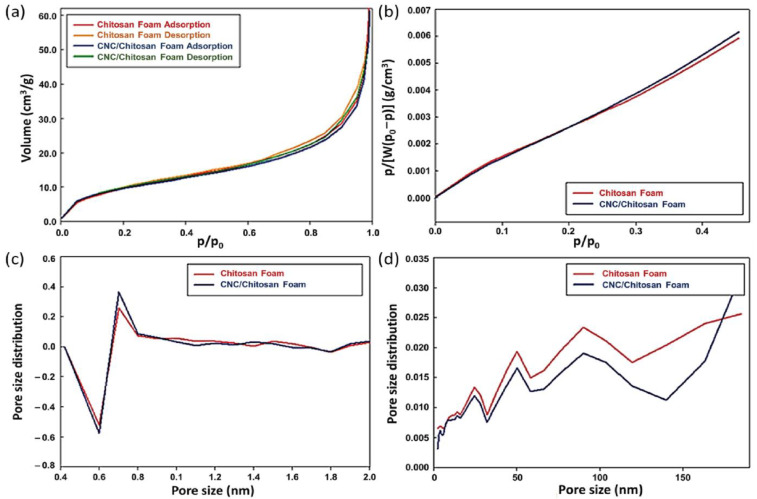
Gas physisorption result of the uncarbonized chitosan foam and CNC/chitosan foam: (**a**) hysteresis loops; (**b**) BET analysis of adsorption isotherm and pore size distributions obtained from (**c**) MP and (**d**) BJH analyses.

To analyze the porous structure, adsorption and desorption analyses were carried out. BET depends on the assumption that adsorption energy is independent of adsorption sites. The BET equation relates the monolayer capacity as follows:

(2) $$\frac{1}{W\left(\frac{P_{0}}{P}-1\right)}=\frac{1}{W_{m}C}+\frac{C-1}{W_{m}C}\left(\frac{P}{P_{0}}\right)$$

where W is the mass of gas adsorbed as monolayer at a relative pressure $P/P_{0}$, P is the actual vapour pressure of adsorbate, $P_{0}$ is the saturated vapour pressure, C is the BET constant, and $W_{m}$ is the required mass of gas adsorbed in a complete monolayer. Figure 6 shows the result of sample gas physisorption before carbonization, and Table 3 lists related calculations. Gas physisorption is an experimental technique based on the Van der Waals interaction between gas molecules and solid particles. Figure 6a presents the hysteresis loops of the samples. Depending on the particle structure, this obtains the different loops. For instance, a porous material with a solid adsorbent–adsorbate interaction offers Langmuir isotherm (i.e., steep uptake at low pressure), implying a micropore structure. The adsorption and desorption curves of the chitosan and CNC/chitosan foams found characterize reversible isotherms for mesopore/macropores with layer structures. For the BET adsorption isotherm analysis, the linear relationship in the relative pressure range from 0.00 to 0.40 was employed (Figure 6b). The SSAs of the chitosan and CNC/chitosan samples were 372.98 and 360.17 m^2^/g, respectively. The samples had pore diameters of 1.21 and 1.19 nm, respectively. Figure 6c,d show that the specimen’s structure was analyzed using the micropore (MP) and BJH analyses. The pore size distribution was obtained based on the physisorption equilibrium isotherms. The BJH analysis considers the pore radius of the adsorption layer, meniscus radius, and thickness. In the chitosan foam case, the micropore volume ratio to mesopore volume was 23.5% versus 82.5%. In the CNC/chitosan foam, the ratio was 20.6% versus 78.4%. These results reveal that both the specimens possessed primarily mesopore, and the CNC composite showed a higher portion of micropore than the chitosan foam.

**Figure 7:**
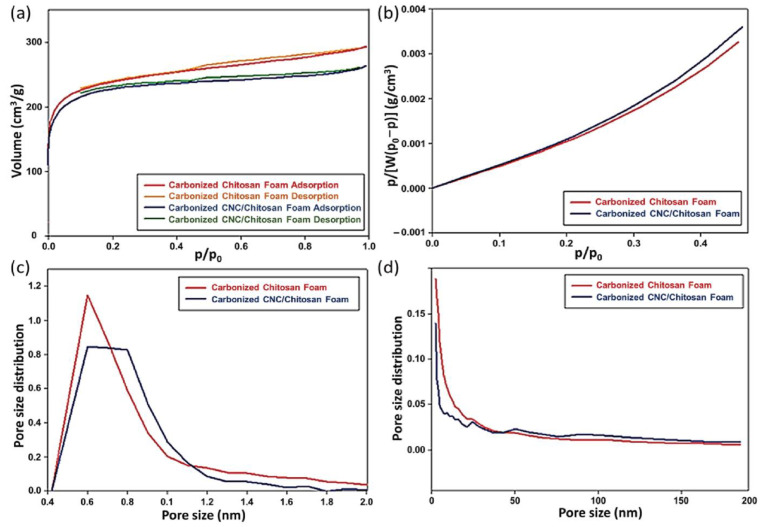
Gas physisorption result of the carbonized chitosan foam and CNC/chitosan foam: (**a**) hysteresis loops; (**b**) BET analysis of adsorption isotherm and pore size distributions obtained from (**c**) MP and (**d**) BJH analyses.

Figure 7 presents the results of the microstructural analysis for the carbonized samples. Table 4 shows the difference between carbonization obtained from the specific surface area and total pore volume, indicating a significant increase. The carbonized specimens showed the Langmuir isotherms (Figure 7a,b). This indicates that the samples mainly possess a microporous structure. The carbonized chitosan and CNC/chitosan foams had 891 and 842 m^2^/g, respectively. The cellulose nanocrystal (CNC)-modified chitosan composite foam showed a slightly smaller pore size than the carbonized chitosan due to the change of crystal structures. The pore diameters for the samples were 2.03 and 1.933 nm, respectively. The pore diameters can affect the electron movement and cyclic voltammetry according to the electrical analysis. Figure 7c,d present the results obtained through the MP and BJH analyses, respectively. The carbonized chitosan foam showed that the ratio of micropore volume to mesopore volume was 75% versus 25%. In the case of the carbonized CNC/chitosan foam, the ratio was 81% and 19%. The carbonization process was found to increase the portion of the micro-pore significantly. In addition, the addition of CNC increased the micropore content in the foam. Overall, we envision the carbonized CNC/chitosan foam as a porous power generator and functional material for biological energy harvesting applications.

## 4. Conclusions

In this study, we investigated carbonized CNC-filled chitosan foam. In order to obtain the CNC nanoparticles, MCC was chemically treated using sulfuric acid to prepare the modified chitosan structures. The NCC was suspended in the chitosan solution, and a freeze-drying process formed the foam structure by evaporating the solvent. After the measurements, the content of 2 wt% NCC in the chitosan foam showed excellent mechanical and electrical properties due to the most appropriate crystal structures. The chemical and structural characteristics of carbonized CNC-embedded CNC/chitosan foam were analyzed through FT-IR and WAXS measurements. The degree of crystallization was modified after the CNC treatment and increased the electron movement and cyclic voltammetry. Compared to CNC/chitosan foam, the carbonized composite foam has a relatively larger crystallite size and higher crystallinity, which had a negative effect on the electrical property. The cyclic voltammetric result indicates that the sample with a vertical structure had more electrochemical performance than the sample with a horizontal structure. In addition, the BET result shows that the carbonized CNC/chitosan showed 81% micropore and total pore size, which was a significant increase compared to the non-carbonized sample. The carbonized CNC/chitosan has an improved internal structure and promotes electro mobility, making it a promising electrode for energy harvesting applications. Furthermore, carbonized composite foams have the potential for porous sensors and actuators in biomedical and environmental areas.

## Figures and Tables

**Table 1:** Raman spectrum result for the carbonized chitosan foam and CNC/chitosan foam.

Materials	Peak		*I_D_/I_G_*
Carbonized Chitosan	D band	1353.1237	1.1600
	G band	1587.2168	
Carbonized CNC/Chitosan	D band	1338.3350	1.1560
	G band	1588.6597	

**Table 2:** WAXS spectrum result of the carbonized chitosan foam and CNC/chitosan foam.

Materials	Crystal Size (nm)	Crystallinity (%)	Interplanar Distance (nm)
Carbonized Chitosan	0.0166	14.0982	0.1537
Carbonized CNC/Chitosan	0.0125	23.9901	0.0147

**Table 3:** Result of BET, MP, and BJH for the uncarbonized chitosan foam and CNC/chitosan foam.

	Materials	Chitosan	CNC/Chitosan
BET	Specific surface area (m^2^/g)	372.9814	360.1709
	Total pore volume (cm^3^/g)	0.1256	0.1142
	Average pore volume (nm)	1.2050	1.1866
MP	Micropore volume (cm^3^/g)	0.2141	0.2021
	Micropore/Total volume	0.4288	0.4107
BJH	Mesopore volume (cm^3^/g)	0.0672	0.0453
	Mesopore/Total volume	0.1622	0.1398

**Table 4:** Result of BET, MP, and BJH for the carbonized chitosan foam and CNC/chitosan foam.

	Carbonized Materials	Chitosan	CNC/Chitosan
BET	Specific surface area (m^2^/g)	890.66	842.37
	Total pore volume (cm^3^/g)	0.4531	0.4072
	Average pore volume (nm)	2.0349	1.9337
MP	Micropore volume (cm^3^/g)	0.4034	0.3767
	Micropore/Total volume	0.8900	0.9250
BJH	Mesopore volume (cm^3^/g)	0.1335	0.0883
	Mesopore/Total volume	0.2950	0.2170

## Data Availability

Data will be made available upon reasonable request to the corresponding author.
