# Correction: Ahn et al. Cellulose Nanocrystal Embedded Composite Foam and Its Carbonization for Energy Application. *Polymers* 2023, *15*, 3454

**DOI:** 10.3390/polym17233208

**Published:** 2025-12-02

**Authors:** So Yeon Ahn, Chengbin Yu, Young Seok Song

**Affiliations:** 1 Department of Fiber Convergence Materials Engineering, Dankook University, Jukjeon-dong, Yongin 16890, Republic of Korea; soyeon@dankook.ac.kr; 2 Research Institute of Advanced Materials (RIAM), Department of Materials Science and Engineering, Seoul National University, Seoul 08826, Republic of Korea

## Error in Figure

In the original publication [1], there was a mistake in Figure 2 as published. The corrected Figure 2 appears below. The authors state that the scientific conclusions are unaffected. This correction was approved by the Academic Editor. The original publication has also been updated.

## Figures and Tables

**Figure 2:**
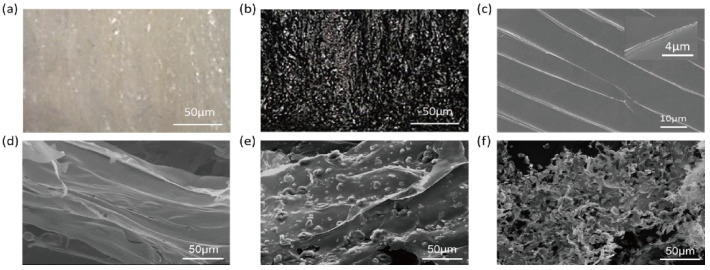
Morphological analysis of the samples: Optical microscopic images of (**a**) the CNC/chitosan foam and (**b**) the carbonized CNC/chitosan foam. (**c**) Cryo-SEM image of (**c**) the CNC/chitosan suspension and SEM images of (**d**) the carbonized chitosan foam, (**e**) the CNC/chitosan foam, and (**f**) the carbonized CNC/chitosan foam.
